# Editorial

**DOI:** 10.1111/1751-7915.12598

**Published:** 2017-01-04

**Authors:** Antoine, Auxi, Juan Luis, Larry, Ken, Siegfried, Willem

Dear microbial biotechnologists and other friends,

2016 was an exciting year for Microbial Biotechnology. As you may have seen, the IF‐2015 of MBT was almost 30% higher than its IF‐2014, advancing from 3.08 to 3.99 and thereby leapfrogged over those of its peers. While IFs can be somewhat ephemeral, the second bit of news we wish to share with you, namely that MBT experienced a 60% increase in submissions in 2016 vis‐a‐vis 2015, encouraged the Editors to raise the originality–significance threshold for acceptance, so we are optimistic that the IF can be pushed ever higher.

One of the major reasons why MBT has been able to position itself ahead of the pack of journals publishing primary research papers in applied microbiology is the outstanding scientists of the Editorial Board, who submit some of their best papers to the Journal, provide rigorous and fair reviews of submitted papers and act as beacons that attract excellent work from others in the field. It is with great pleasure that we express here our deep gratitude to the Editorial Board for its dedication, loyalty and hard work.

The vision of the MBT Editor Team is not only for the Journal to be the premier vehicle for very best work in microbial biotechnology, but also to actively promote new, pioneering and game‐changing topics that will have a major impact on, and determine the future trajectory of, the field. One exercise we undertook in 2016 was the publication of a Special Issue designated ‘Microbial Biotechnology‐2020’, whose brief was, in the framework of grand challenges, to define where we want to be in 2020, articulate the obstacles to getting there and propose a road map for dealing with the obstacles and arriving at the goal(s). These articles are, we think, outstanding and stimulating, and we hope you enjoyed reading them. Related to this, but different, is the biennial Crystal Ball feature which appears in this issue, in which authors speculate on the nature of new developments that may bring about a step change in progress in the field. As always, these CBs will be one of the most enjoyed features of the Journal.

One of the most exciting developments in recent years has been the realization that we and other beings on the planet are not only covered by microbes, but that they contribute in a pivotal manner to our physiology and metabolism: while it may be true that ‘we are what we eat’, we are also what our microbiome determines. While the early days of microbiome research largely involved figuring out who our microbial partners are and what they do, the field is now beginning to discover, envision and develop novel and ingenious microbiome applications. This will inevitably lead to the creation of start‐ups and new divisions within established commercial enterprises, and resulting opportunities for employment of young microbiome scientists. MBT aims to be a primary vehicle for publications reporting microbiome applications. As part of this new initiative, this year we will regularly feature Editorials on the topic of potential areas of business opportunities–job creation emerging from microbiome research. Contributors will include *inter alia* Jack Gilbert, Harald Bruessow, Victor de Lorenzo and Braj Singh. The first of these, on microbiome transplants, by van der Lelie, Taghavi, Henry and Gilbert, appears in this issue, is inspiring and eloquently testifies to the new commercial opportunities of this paradigm change in our perception of what we are.

There were changes in the Editor Team in 2016: Auxiliadora Prieto replaced Marty Rosenberg, who cycled off (thank you so much Marty, for all the terrific contributions you made over the formative years of the Journal!), and Antoine Danchin became Editor of our Genomics Update Feature. Both are exceptional leaders in their respective fields and bring valued experience and skills that substantively enrich the team.

Finally, we reiterate something we have stated before: the MBT Editor Team is inclusive in its philosophy and welcomes ideas and suggestions for improvement from its readership. If you do have a suggestion, please communicate it to one of us and we will consider it.

On that note, we wish you all a happy and healthy year 2017, with important successes that you report in our favourite Journal!

